# GRB10 is a novel oncogene associated with cell proliferation and prognosis in glioma

**DOI:** 10.1186/s12935-022-02636-5

**Published:** 2022-07-05

**Authors:** Yuanbing Chen, Miao Tang, Jianbing Xiong, Qiongjue Gao, Wuyang Cao, Jun Huang

**Affiliations:** 1 Department of Neurosurgery, Xiangya Hospital, Central South University, Changsha, 410008 Hunan China; 2 National Clinical Research Center for Geriatric Disorders, Xiangya Hospital, Central South University, Changsha, 410008 Hunan China; 3 Department of Emergency, Xiangya Hospital, Central South University, Changsha, 410008 Hunan China; 4 Department of Acupuncture and Rehabilitation, The Second Affiliated Hospital, Hunan University of Chinese Medicine, Changsha, 410005 Hunan China; 5 Department of Neurosurgery, The First People’s Hospital of Changde City, Changde, 415003 Hunan China

**Keywords:** Glioma, GRB10, Prognosis

## Abstract

**Background:**

Glioma is the most common malignant tumor of the central nervous system and is associated with a poor prognosis. This study aimed to explore the function of growth factor receptor-bound protein 10(GRB 10) in glioma.

**Methods:**

The expression of GRB10 in glioma was determined based on the glioma transcriptome profile downloaded from The Cancer Genome Atlas (TCGA), Chinese Glioma Genome Atlas (CGGA), and Gene Expression Omnibus (GEO) databases. RT-qPCR was performed to detect the expression of GRB10 in tissue samples obtained from 68 glioma patients. The patients were followed up via telephone or in-person outpatient visits to determine survival. Kaplan-Meier survival analyses were used to evaluate the effect of GRB10 on the prognosis of glioma patients. Further, we constructed GRB10 knockdown cell lines were constructed to investigate the effect of GRB10 on glioma. The cell growth, colony formation, cell cycle assay, EdU assay, and tumor formation in xenograft were performed.

**Results:**

The expression level of *GRB10* was positively correlated to the histological grades of gliomas. In addition, Kaplan-Meier survival curves revealed that glioma patients with lower expression of *GRB10* had more prolonged survival. The knockdown of GRB10 was shown to inhibit cell proliferation, colony formation, and tumor formation in the xenograft models. Cell cycle assay revealed that the knockdown of GRB10 can inhibit the cells entering the G2/M phase from the S phase. The analysis of GSEA suggests that the expression of *GRB10* was positively correlated with the hypoxia and EMT signaling pathway.

**Conclusions:**

Our data revealed that GRB10 regulated tumorigenesis in glioma and played a vital role in promoting the glioma progression, which indicated that GRB10 could be used as a potential prognostic marker.

**Supplementary Information:**

The online version contains supplementary material available at 10.1186/s12935-022-02636-5.

## Background

Glioma is the frequently occurring malignancy neoplasm in the central nervous system. Glioblastoma multiforme (GBM) is the most aggressive glioma, which is still untreatable with a poor median survival of 14.6 months despite advances in treatment modalities, including surgery, radiation, chemotherapy, and targeted therapy [1–3]. Several molecular markers and related molecular mechanisms for glioma were identified in the last decades. However, these markers were not associated with prognostic significance [4–6]. Thus, it is essential to detect novel markers to predict prognosis and adjuvant treatment for glioma.

The growth factor receptor-bound protein 10(GRB10) plays a vital role in regulating insulin-receptor signaling. The GRB10 is also identified as a diabetes susceptibility locus [7–10]. GRB10 has been shown to play a tumorigenesis role in rapidly proliferating tumors. For instance, the expression of GRB10 is upregulated in acute myeloid leukemia (AML), which is associated with increased cell cycle progression and cell proliferation. In addition, it is associated with poor overall survival [11–13]. GRB10 is also involved in prostate cancer tumorigenesis [14], and is upregulated in cervical squamous carcinoma [15]. However, GRB10 has been reported to be downregulated in some solid tumors [16]. Therefore, further research is required to reveal the function of GRB10 in oncogenesis and tumor progression in the different tumor types.

GRB10 is one of the GRB7 protein family members (GRB7, GRB10, GRB14) [17]. A previous study revealed that GRB7 promoted the development and angiogenesis of gliomas [18]. However, the role of GRB10 and GRB14 in glioma has not been fully elucidated. This study aims to investigate the role of GRB10 in glioma. Further, the study aims to conduct *in vitro* and *in vivo* assays to elucidate the functions of GRB10 in the development of glioma.

## Materials and methods

### Bioinformatics prediction

The transcriptome profile of glioma was downloaded from The Cancer Genome Atlas (TCGA), Chinese Glioma Genome Atlas (CGGA), and Gene Expression Omnibus (GEO) databases (GSE 43,378). Further, the expression of GRB10 was analyzed in different tumor grades. Survival analysis was determined using the Kaplan-Meier method, and the patients were divided into high and low expression groups according to the median of the expression of GRB10.

The GSEA was used to analyze the differentially enriched pathway between the high and low expression of GRB10, which based on the patients were divided into GRB10 high and low groups according to their median, and the expression profile of GRB10 was obtained from TCGA and CGGA. The limma for the R platform was performed to visualize the differentially expressed mRNAs of *GRB10* between the high and low expression of *GRB10.*

### Patients in the study

Tumor tissues were obtained from 68 patients without any treatment before surgery, which were stored in the refrigerator at -80 °C, including 42(61.8%) males and 26(38.2%) females, aged 1–70 years (median age of 47.91 ± 1.564 years) with primary glioma who had received radical resection between March 2017 and January 2021 at the Xiangya Hospital. The patients were followed-up by telephone or during in-person outpatient visits. The gliomas were graded according to the WHO classification system as grade II (n = 20), grade III (n = 16), grade IV (n = 32), and peritumor tissue (n = 13). This study was approved by the Research Ethics Committee of the Xiangya hospital.

### Cell culture, plasmids, and shRNAs

The gliomas cells U251 and SHG44 were cultured in DMEM medium (Gibco, #C1995500BT, Switzerland) supplemented with 10% fetal bovine serum (FBS, Sigma, #12103C, USA). These cell lines were maintained at 37 °C in a humidified atmosphere containing 5% CO_2_.

Lentiviral shRNA clones targeting human GRB10 and the nonspecific target control (GV248) were purchased from GeneChem (Shanghai, China). According to the manufacturer’s protocol of Lipofectamine^®^ 2000, lentiviral particles were produced in 293 T cells. U251 and SHG44 cell lines pre-seeded in six-well plates and transfected with lentiviral particles accomplished 10 mg/ml polybrene. Stably expressed colonies were selected with 2 μg/ml of puromycin. The sequences of shGRB10#1: GCCGCAAAGCAGGATGTTAAA, shGRB10#3: ATGCTCCTTTACCAGAATTAC.

### RNA isolation and quantitative real-time PCR (RT-qPCR)

Total RNA was extracted using RNAiso Plus (Takara, #9109, Japan). According to the manufacturer’s protocol of PrimeScript™RT reagent Kit with gDNA Eraser (Takara, #RR047A, Japan), the gRNA was isolated and a quantity of 1 μg gRNA reverse transcribed into cDNA. The RT-qPCR was performed by using Fast Start Universal SYBR Green Master (Roche, #4913914001, Switzerland) in a 7500 Fast Real-Time PCR System (Applied Biosystems, Life Technologies, USA). The relative gene expression levels were normalized to β-actin. The following primers: GRB10 forward sequence: AGGACACAGCACTGGTTTCACG and reverse sequence: TCTGGCTGTCACGGAGGAGAAA. β-actin forward sequence: CACCATTGGCAATGAGCGGTTC and reverse sequence: AGGTCTTTGCGGATGTCCACGT.

### Western blot (WB)

Glioma cells were precipitated and lysed in immunoprecipitation (IP) lysis buffer. The quantity of 30 mg total protein was electrophoresed in 10% SDS-PAGE gels and transferred onto polyvinylidene fluoride (PVDF). These membranes were incubated with primary antibodies against GRB10(Proteintech, #23,591-1-AP, 1:1000, USA), and β-actin (Sigma, #A5441, 1:10000, USA). Subsequently, the membranes were incubated for 1 h at room temperature with the secondary antibodies. The membranes were then washed three times with PBST. Chemiluminescent signals were determined using the ChemiDox XRS+ imaging system (Bio-Rad, USA).

### Cell proliferation assays, migration and invasion assays, colony formation assays

The glioma cells were precipitated by centrifugation and counted. Cells were seeded into 96 well plates at a density of 500 cells/well in complete medium (200 μl) supplemented with 10% FBS. Cell viability was determined using CellTiter 96^®^ AQueous One Solution Cell Proliferation Assay (Promega, #G3588, USA).

The effect of GRB10 on cell migration ability was determined using the transwell assay. Cells at a density of 1 × 10^4^ were seeded onto transwell inserts containing medium supplemented with 1% FBS(200 μl). Further, the transwell inserts were placed into 24-well plates containing 800 μl complete medium supplemented with 10% FBS. After incubation for 24 h, cells that migrated to the bottom of the transwell inserts were fixed and stained with crystal violet. The migrated cells were visualized using a microscope.

Cell invasion was determined by the Matrigel invasion assay. The transwell inserts were uniformly coated with 100 μl Matrigel basement membrane matrix (medium: Matrigel = 8:1, BD, #356230, USA) for two hours. The transwell inserts were then seeded with a total of 1 × 10^5^ cells in 200 μl medium containing 1% FBS, and placed into 24-well plates added with 800 μl complete medium containing 10% FBS. After incubation for 48 h, the cells that had invaded the bottom of the transwell inserts were fixed, stained with crystal violet, and counted using a microscope.

Furthermore, 500 cells were seeded into six-well plates containing a complete medium. The cells were incubated for 14 days at 37 °C in a humidified atmosphere containing 5% CO_2_ to form colonies. Finally, the colonies were fixed with methanol for ten minutes and stained with crystal violet for 15 min. The colonies were captured and counted by Image J.

### 5-Ethynyl-2’-deoxyuridine (EdU) assay

Cells were seeded into a 24-well plate overnight, followed by incubation with Cell-Light EdU Apollo488 in Vitro Kit (RIBOBIO, #C10310-3, China) for 2 h. 4% formaldehyde was used to fix the cell after washing with PBS twice. Subsequently, glycine (2 mg/ml) (Sangon Biotech, #56-40-6, Shanghai, China) was added for 5 min, and 0.5% Triton-X-100(Sangon Biotech, #9002-93-1, Shanghai, China) was added into cells for 10 min incubation. Then, cells were treated with Apollo for 30 min and washed with 0.5% Triton-X-100 three times. Next, the cells were stained with Hoechst 33342 and protected from light for 30 min at room temperature. Cell proliferation was assessed by Fluorescence Microplate Reader (BioTek).

### Wound healing assay

Cells were cultured in a 6-well plate overnight. A scratch was performed in the cell monolayer using a 200 ml pipette tip, washed with PBS twice, and the completed medium was added. Images 20X of the wound area were captured at 0 and 24 h by EVOS. The relative migration rate was calculated in three fields of each experimental condition.

### Cell cycle

The glioma cells were harvested and counted. According to the manufacturer’s protocol of Cell Cycle Staining Kit (MultiScience Biotech, #CCS01, China), cells were stained with DNA Staining solution(1 ml) and Permeabilization solution (10 μl) for 30 min. Stained cells (5 × 10^5^cells/mL) were identified by using a flow cytometer (BD Biosciences, USA). The percentages of cells in the G1, G2/M, and S phases (data from 20,000 events per sample) were calculated by Flowjo.

### Animal experiments

Tumorigenesis was evaluated using the xenograft tumor formation assay as previously described [19, 20]. Four-week-old male BALB/c athymic mice were purchased from the Hunan SJA Laboratory Animal Co. Ltd, a total of 15 mice were randomly divided into three groups (5 mice in each group). The mice were housed in specific pathogen-free barrier conditions. The glioma cells were precipitated by centrifugation and counted. After that, a total of 5 × 10^5^ cells in 100 μl PBS were injected subcutaneously into the mice. Tumor length and width were measured every 3 days. The tumor volume was determined as: tumor volume (mm^3^) = 1/2 × (tumor length) × (tumor width)^2^.

All the mice were euthanized on day 28 by inhalation of carbon dioxide. Briefly, the mice were placed in a closed transparent chamber. After that, CO_2_ was constantly injected into the chamber at a rate of 30–50% per minute until the mice died. Death was confirmed by observation for five minutes. All animal experiments were approved by the Institutional Animal Care and Use Committee of the Central South University of Xiangya School of Medicine. In addition, the procedures conformed to the legal mandates and federal guidelines for the care and maintenance of laboratory animals.

H&E staining: Tissues from mice were prepared and fixed in Formalin for 24 h and transferred to 70% ethanol. H&E staining was performed.

### Statistical analysis

The IBM SPSS Statistical 21.0 and GraphPad Prism 8.0 software were used for statistical analysis. Results are shown as the mean ± SEM or SD. A Pearson χ^2^ test was applied to compare the categorical variables between the group. The statistical differences between two the groups were analyzed by an unpaired Student’s *t*-test (two-tailed) if data were normally distributed. Otherwise, data were analyzed by Mann–Whitney test. Survival analysis was determined using the Kaplan-Meier method. *p-*values < 0.05 were considered statistically significant, *p < 0.05, **p < 0.01, ***p < 0.001, and ****p < 0.0001.

## Results

### The mRNA expression levels of GRB10 in the TCGA, CGGA, and GEO datasets

**Fig. 1:**
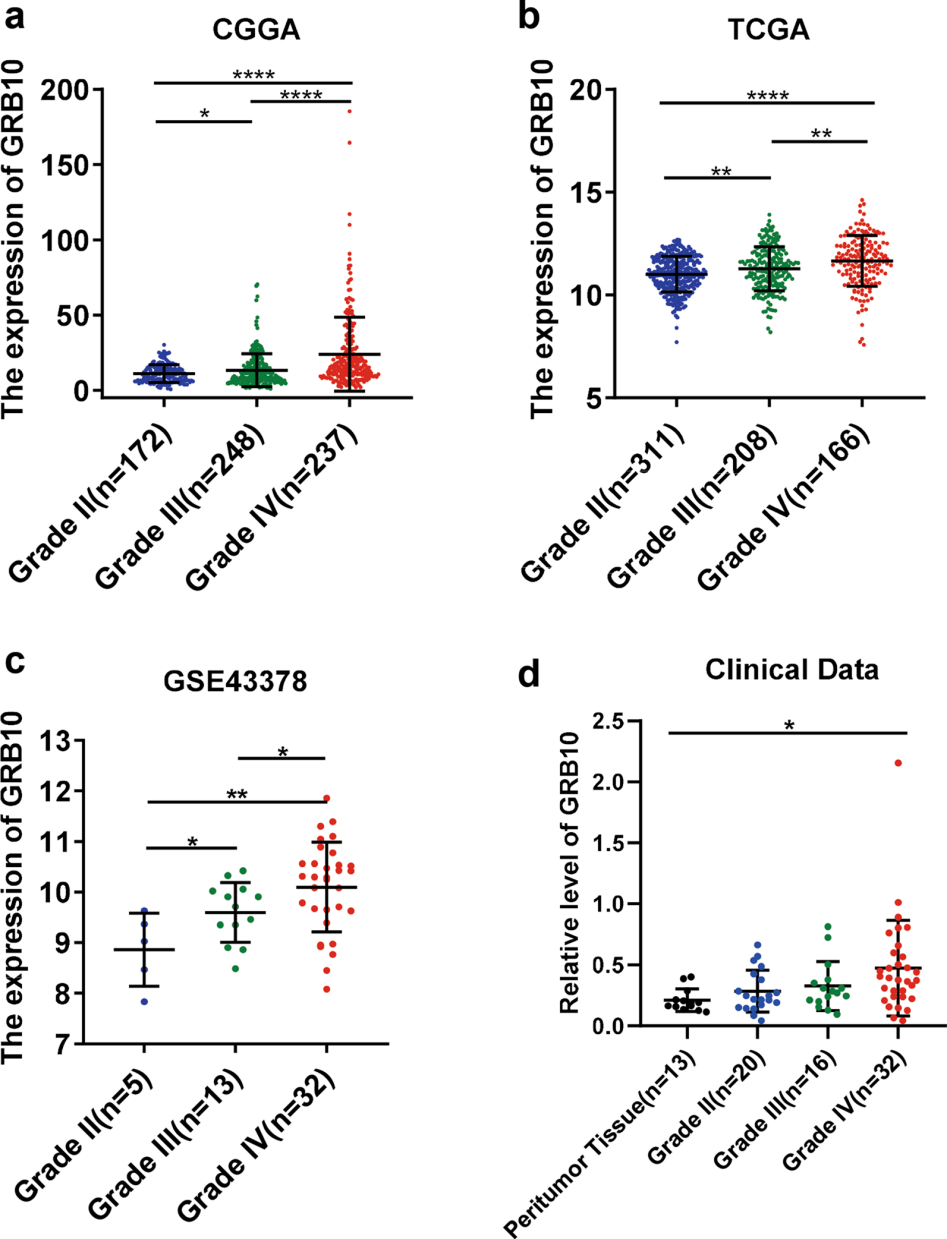
The mRNA expression levels of GRB10 in the TCGA, CGGA and GEO dataset. **a**-**c** The expression of GRB10 in different histological grades was analyzed based on the gene expression profile data, including CGGA (**a**) (grade II, n = 172; grade III, n = 248; and grade IV, n = 237), TCGA (**b**) (grade II, n = 311; grade III, n = 208; and grade IV, n = 166), GEO (**c**) (grade II, n = 5; grade III, n = 13; and grade IV, n = 32). **d** qRT-PCR was used to evaluate the expression of GRB10 in tumor tissues (peritumor tissue, n = 13; grade II, n = 20; grade III, n = 16; and grade IV, n = 32). Data are analyzed by Mann–Whitney test. *p < 0.05, **p < 0.01, ***p < 0.001, and ****p < 0.0001

The mRNA expression of *GRB10* in different histological grades was analyzed from the TCGA, CGGA, and GEO(GSE43378) databases. The expression of *GRB10* was positively correlated to the tumor grades (Fig. 1a–c). For example, the mRNA of *GRB10* was highly expressed in grade IV gliomas. In addition, the expression of *GRB10* was significantly correlated with patient age, IDH1 mutation, 1p/19q codeletion, and MGMT promoter methylation (Tables 1, 2).

**Table 1:** Characteristics of patients with glioma based on CGGA

Characteristic	Number (%)	GRB10 expression		*P* value
		Low	High	
Gender				0.2519
Female	231(55.9%)	121	110	
Male	182(44.1%)	85	97	
Age(year)				0.0045
< 60	369(89.3%)	193	176	
≥ 60	44(10.7%)	13	31	
Grade				< 0.0001
WHO II	96(23.2%)	57	39	
WHO III	161(40.0%)	95	66	
WHO IV	156(37.8%)	54	102	
IDH status				< 0.0001
Mutant	185(44.8%)	54	131	
Wildtype	228(55.2%)	152	76	
1p19q status				0.0007
Codel	87(21.1%)	51	36	
Non-codel	326(78.9%)	155	171	
MGMT status				0.0393
Methylated	244(59.1%)	132	112	
Un-methylated	169(40.1%)	74	95

**Table 2:** Characteristics of patients with glioma based on clinical samples

Characteristic	Number (%)	GRB10 expression		*P* value
		Low	High	
Gender				
Female	26(38.2%)	11	15	0.3182
Male	42(61.8%)	23	19	
Age(year)				0.2830
< 60	59(86.8%)	31	28	
≥ 60	9((13.2%)	3	6	
Grade				0.0204
WHO II	20(29.4%)	14	6	
WHO III	16(23.5%)	9	7	
WHO IV	32(47.1%)	11	21	

To further demonstrate the expression of GRB10 in glioma, we extracted the RNA from glioma, which was stored in the refrigerator at −80 °C. Similarly, the RT-qPCR analysis revealed that *GRB10* was highly expressed in higher histological grade tumors (Fig. 1d, Table 2). Taken together, the mRNA expression of *GRB10* was positively correlated to the tumor grade, suggesting that GRB10 plays an oncogenic role in gliomas.

### Correlation between GRB10 and patients’ prognosis

**Fig. 2:**
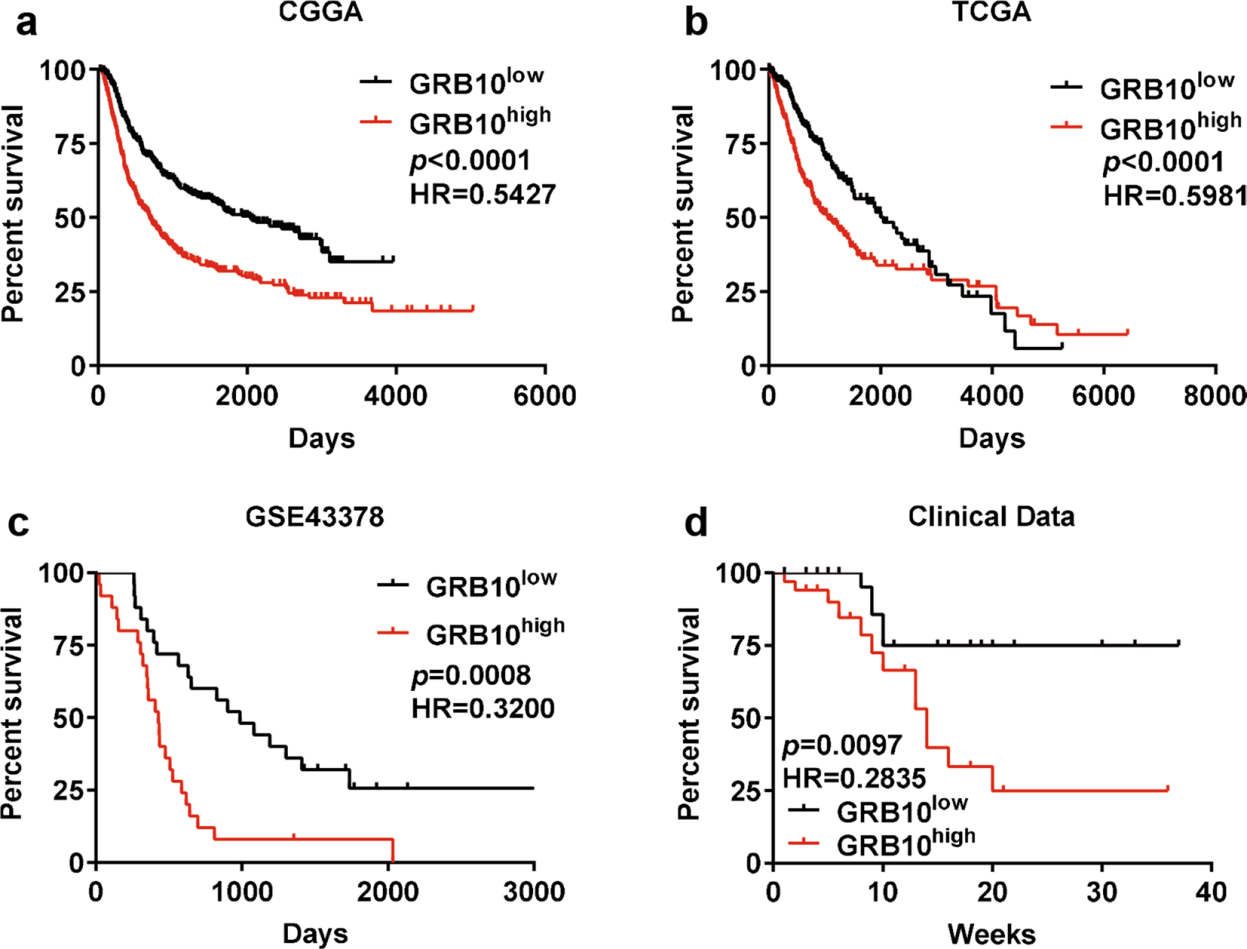
Correlation between GRB10 and glioma patients’ prognosis. Kaplan–Meier analysis revealed overall survival (OS) curves of glioma patients with different expressions of GRB10 in CGGA database (**a**), TCGA database (**b**), GEO database (**c**), and clinical patients (**d**)

The survival analysis was conducted with Kaplan–Meier plots based on the TCGA, CGGA and GEO (GSE43378) database. The results showed that a low expression of *GRB10* was associated with a longer survival period compared with a high expression of *GRB10* (Fig. 2a–c). The mRNA expression level of *GRB10* was evaluated in 68 patients with different grades of gliomas. The patients were followed up to determine survival. Consistently, a high expression of *GRB10* was associated with a shorter OS. There were significant differences in OS between the high GRB10 expression group and the low *GRB10* expression group (Fig. 2d). Taken together, the expression of *GRB10* was correlated to the OS of patients, suggesting that GRB10 performs a critical role in glioma progression.

### Knockdown of GRB10 inhibits glioma cell growth, colony formation, invasion, and migration

**Fig. 3:**
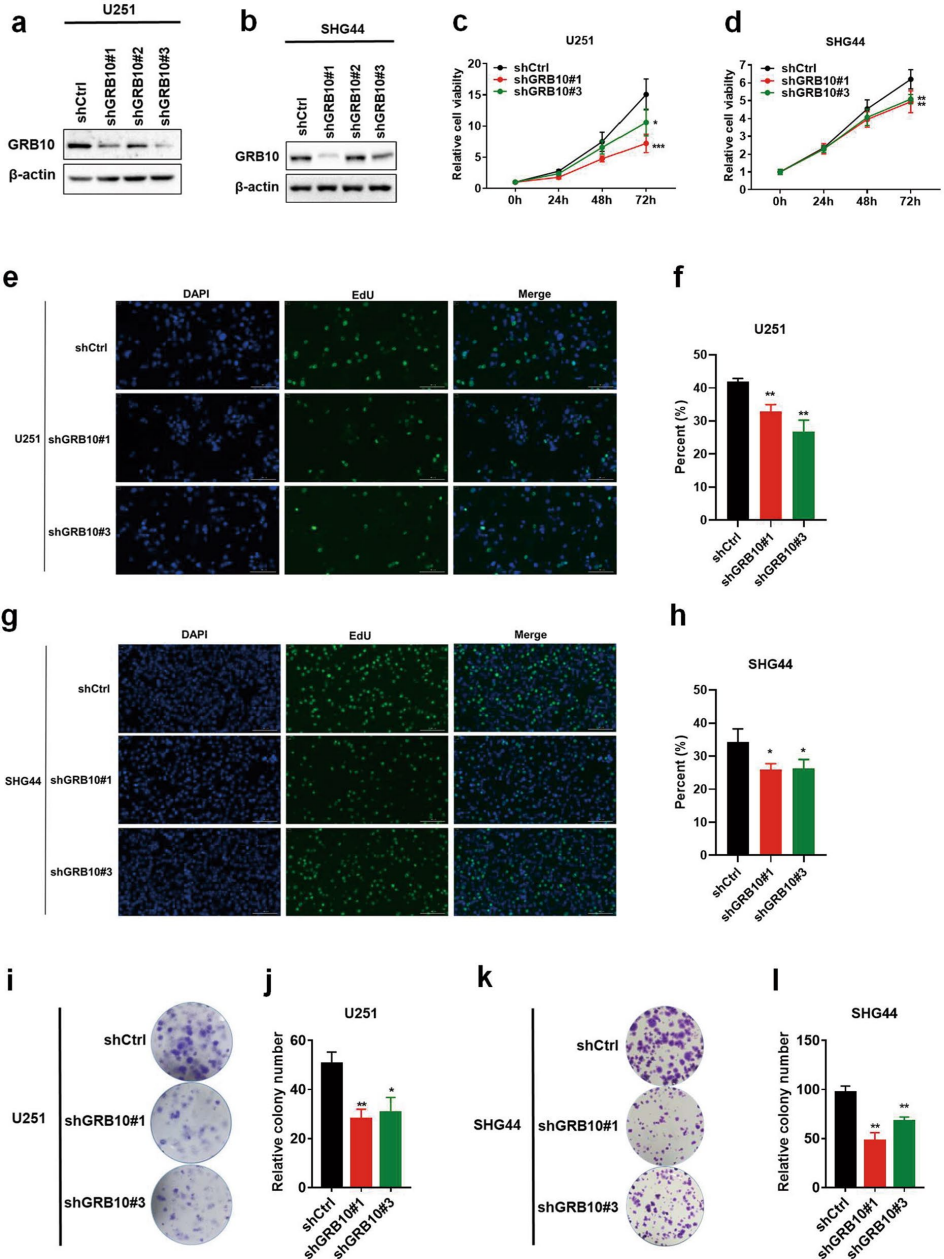
Knockdown of GRB10 inhibits glioma cell growth and colony formation. **a**, **b** The WB was conducted to determine the level of GRB10 in U251(**a**) and SHG44 (**b**) cells stably knockdown of GRB10. **c**, **d** The cell viability detected by MTS assay in U251 (**c**) and SHG44 (**d**) cells stably knockdown of GRB10. **e**–**h** EdU was performed to determine the cell viability in U251 (**e**–**f**) and SHG44 (**g**–**h**) cells stably knockdown of GRB10. **i**–**l** Colony-formation assay of U251 and SHG44 cells with stably knockdown of GRB10 (**i**, **j**), colony number counted by Image J (**k**, **l**). Data are analyzed by a two-tailed Student’s *t-*test. *p < 0.05, **p < 0.01, ***p < 0.001, and ****p < 0.0001

**Fig. 4:**
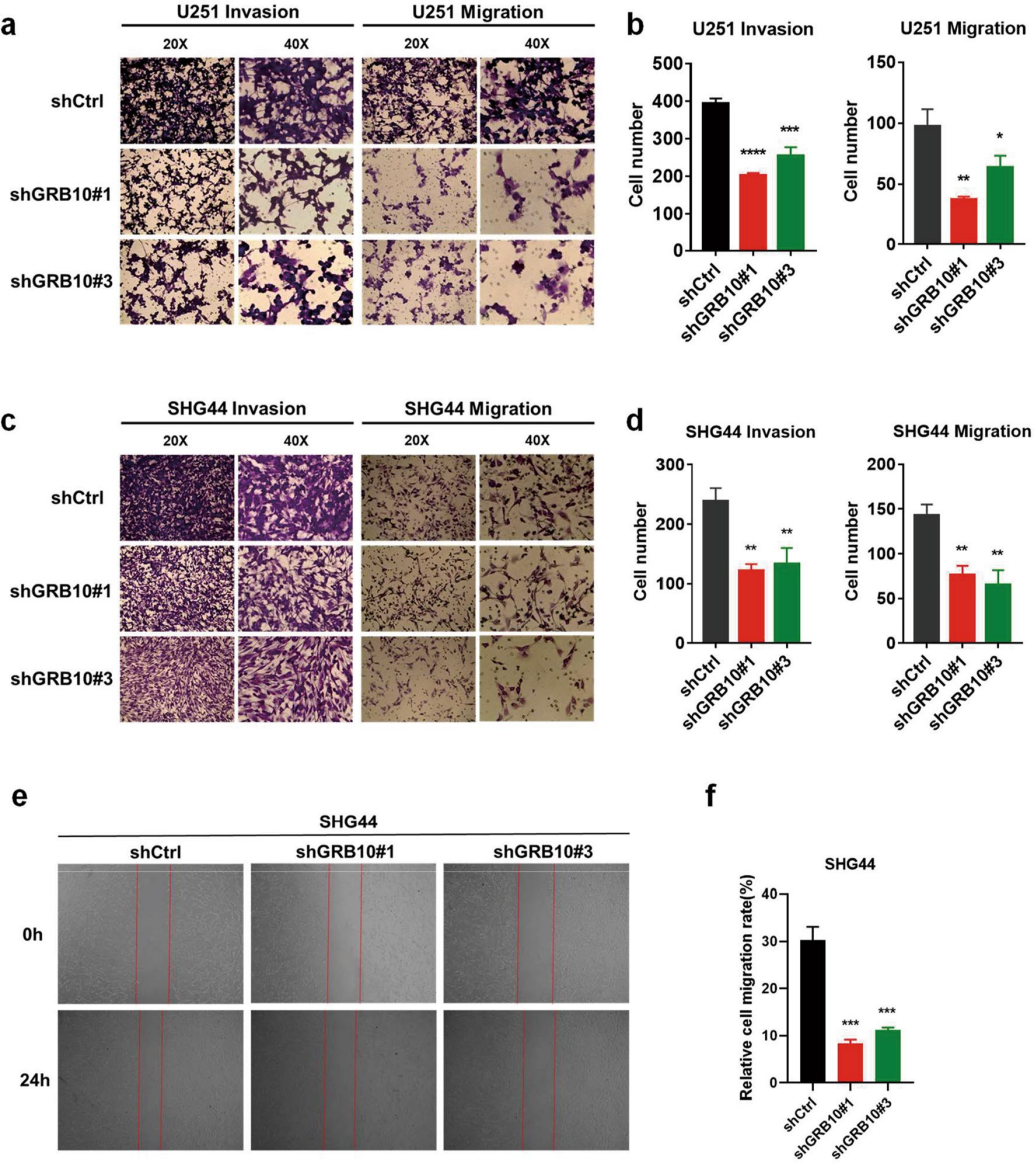
Knockdown of GRB10 inhibits cell invasion and migration in glioma. **a** The transwell migration assay showed that knockdown of GRB10 inhibited migration and invasion of U251. **b** Image J was used to count the cells that had invaded or migrated into the bottom of the transwell inserts. **c** Knockdown of GRB10 was shown to inhibit the migration and invasion of SHG44 cells. **d** Image J was used to count the SHG44 cells that had invaded or migrated into the bottom of the transwell inserts. **e**–**f** Wound healing assay showed that knockdown of GRB10 was inhibit the ability of migration in SHG44 cells. Data are analyzed by a two-tailed Student’s *t-*test. *p < 0.05, **p < 0.01, ***p < 0.001, and ****p < 0.0001

To elucidate the function of GRB10 in glioma, U251 and SHG44 cell lines were stable knockdowns of GRB10 with two separate sequences by using lentivirus, WB performed to detect the efficiency of knockdown (Fig. 3a, b, Additional file 1: Figure S1). To determine whether the silencing of GRB10 affected the ability of proliferation in glioma cells, the MTS and EdU assays were performed. Knockdown of GRB10 in U251 and SHG44 cell lines was shown to inhibit cell growth significantly (Fig. 3c–h). Besides, knockdown of GRB10 was shown to significantly suppress colony formation in the two cell lines (Fig. 3i–l). Further, the transwell assay (Fig. 4a–d) and wound-healing assay (Fig. 4e, f) revealed that knockdown of GRB10 significantly impaired cell invasion and migration of U251 and SHG44. These results showed that the expression of GRB10 affected cell growth, colony formation, invasion, and migration of glioma cell lines.

### Effects of GRB10 on glioma cell cycle

**Fig. 5:**
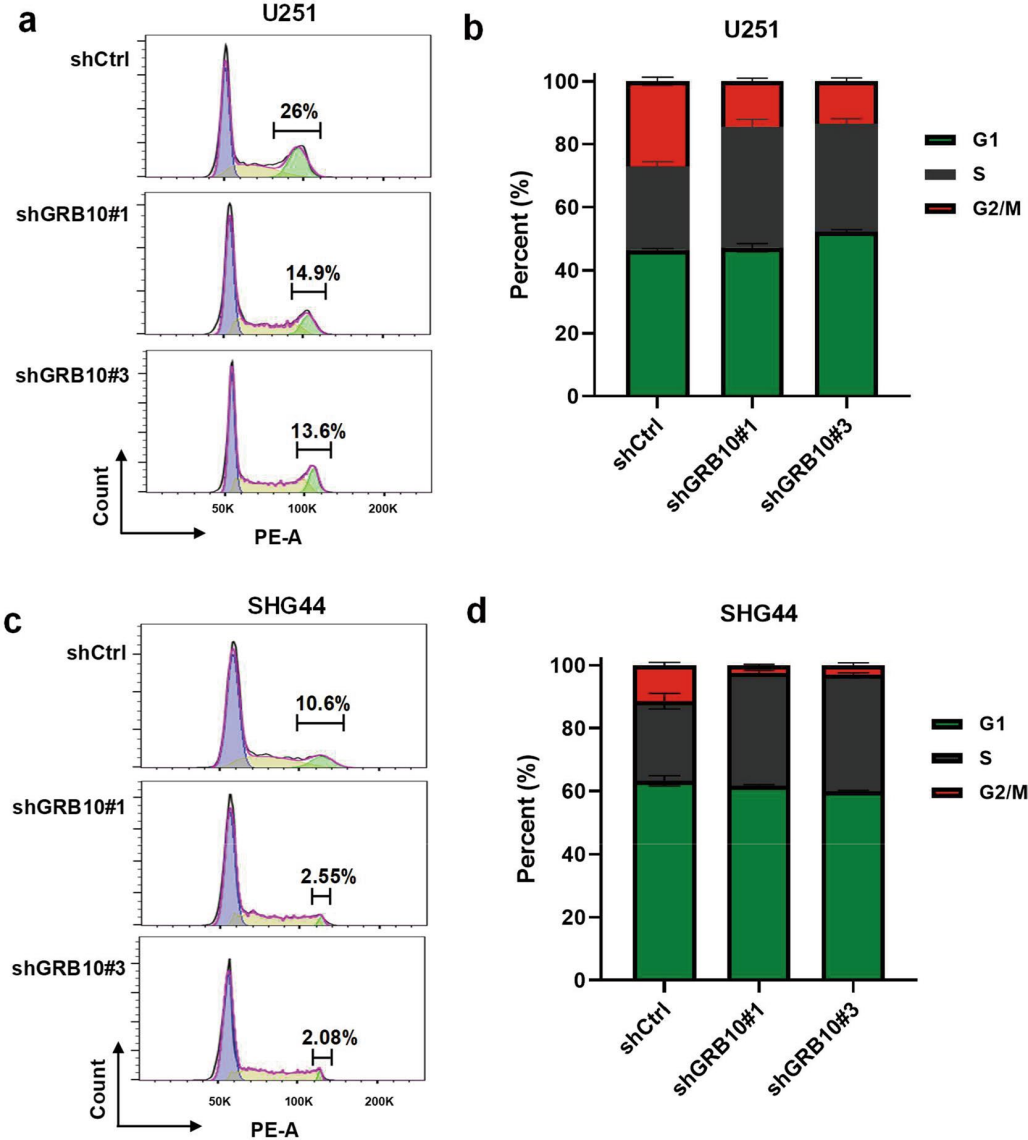
Effects of GRB10 on glioma cell cycle. a–d The flow cytometry determines the effect of GRB10 on the cell cycle in glioma, knockdown of GRB10 inhibited the cells entering the G2/M phase from the S phase in U251 (**a**, **b**) and in SHG44 (**c**, **d**)

Flow cytometry was performed to determine the effect of GRB10 on cell cycle distribution in U251 and SHG44. The results revealed that knockdown of GRB10 inhibited the cells entering the G2/M phase from the S phase both in U251 (Fig. 5a, b) and SHG44(Fig. 5c, d).

### *Knockdown of GRB10 attenuates tumor formation *in vivo

**Fig. 6:**
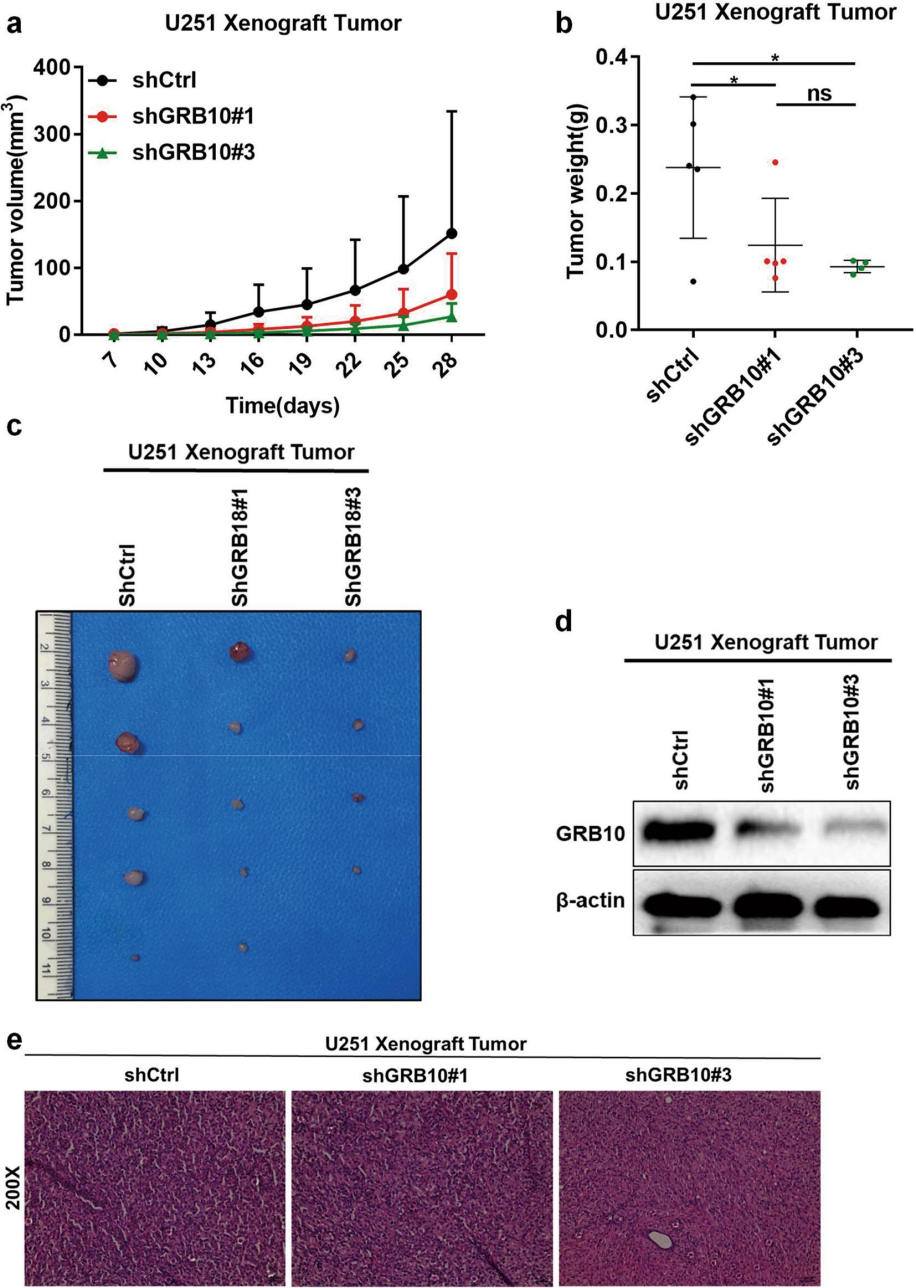
Knockdown of GRB10 attenuates in vivo tumor formation. **a** Tumor volume was measured in nude mice following injection of U251 cells with stably knockdown of GRB10 or the control vector at the indicated time points. **b** Tumor weights were recorded. **c** The harvested xenograft tumors. **d** The expression of GRB10 detected by WB in xenograft tumors. **e** The xenograft tumors were detected by HE. Data are analyzed by a two-tailed Student’s *t*-test. *p < 0.05, **p < 0.01, ***p < 0.001, and ****p < 0.0001

U251 cells were injected subcutaneously into the nude mice to assess tumor formation. The results revealed that depleting the expression of GRB10 reduced the tumor size, tumor volume, and weight significantly (Fig. 6a–e, Additional file 1: Figure S2). Taken together, the expression of GRB10 was positively correlated to tumor growth.

### Potential downstream pathways and target genes of GRB10

**Fig. 7:**
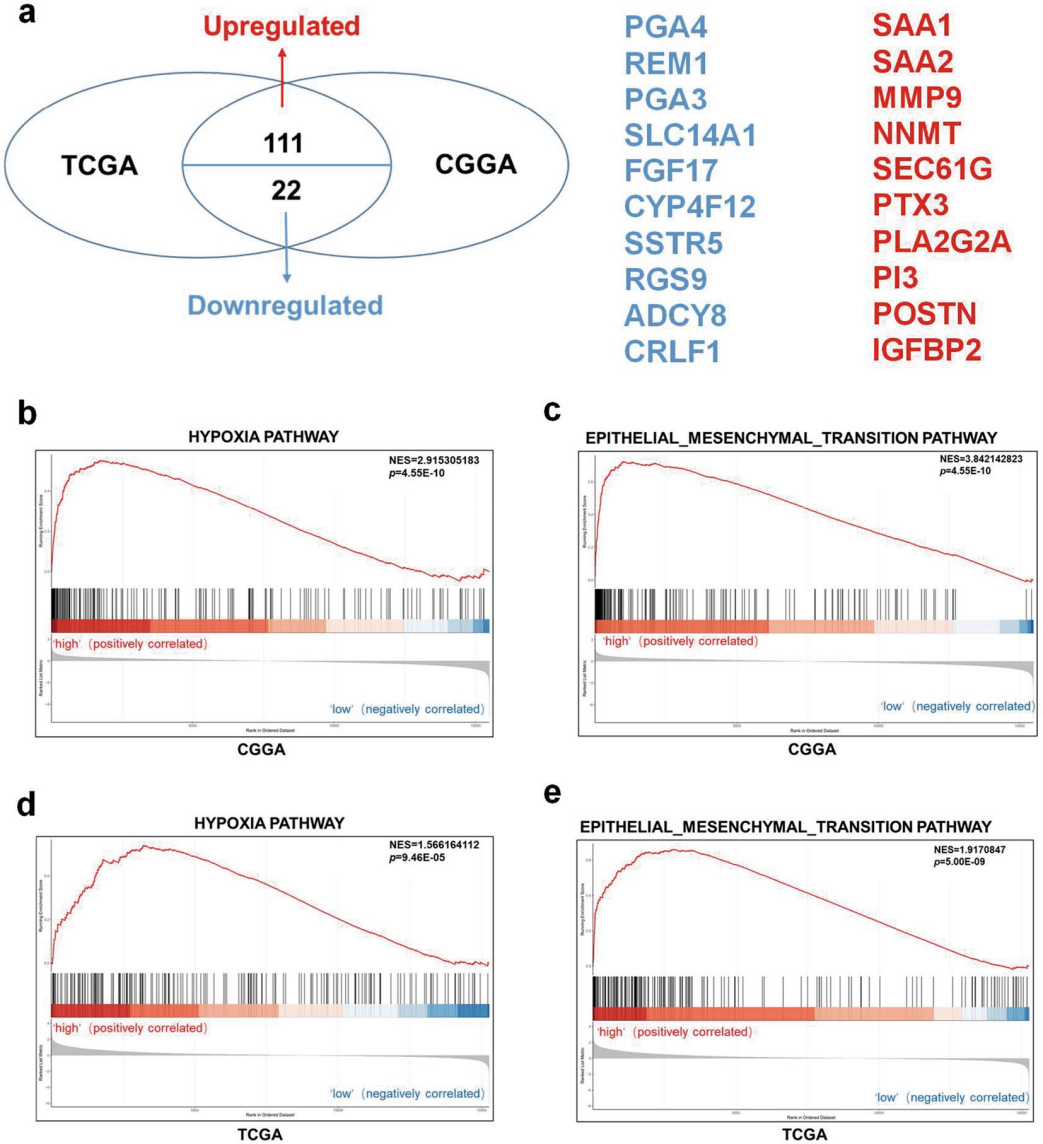
Potential downstream pathways and target genes of GRB10. **a** TCGA and CCGA databases were found to differentially up-regulate 111 genes and down-regulate 22 genes together. The top 10 genes were upregulated in red, and the top 10 genes were downregulated in blue. **b**-**c** GSEA suggests that the expression of GRB10 was positively correlated with the hypoxia (**b**) and EMT(c) in CGGA. **d**-**e** The expression of GRB10 was positively associated with the pathway of hypoxia (**d**) and EMT(**e**) in TCGA

To preliminarily explore the potential downstream pathways and target genes of GRB10, both the TCGA and CCGA databases were divided into the high and low expression of GRB10 according to the expression level of GRB10. The two data were found to differentially up-regulate 111 genes and down-regulate 22 genes together (Fig. 7a). GSEA suggests that the expression of *GRB10* was positively correlated with the hypoxia and EMT signaling pathway (Fig. 7b–e).

## Discussion

According to the classification of the World Health Organization (WHO), gliomas are divided into four grades (I, II, III, IV). Grade I/II gliomas are termed low-grade gliomas. In contrast, grade III/IV gliomas have a high proliferation and invasion ability with a shorter survival period and are classified as high-grade gliomas [21, 22]. In recent years, to improve the exact judgment of prognosis, many studies have focused on finding new biomarkers to predict the prognosis of gliomas [23, 24]. However, the ideal prognosis marker and molecular mechanisms remain further studied in glioma. This study analyzed the gene expression in the TCGA, CGGA, and GEO databases. The expression of GRB10 was shown to be positively correlated with the histological grades of gliomas. Further, RT-qPCR revealed that patients with higher histological tumor grades had a higher expression of *GRB10*. These results suggest that GRB10 is involved in tumorigenesis and tumor progression in gliomas.

Growth factor receptor-bound protein 7 (GRB7) is a functionally multidomain adaptor protein GRB7 also partners with the activated epidermal growth factor receptor (EGFR), which binds to phospho-tyrosine-related oncogenic signaling molecules, thus regulating the oncogenic signal transduction. Further, GRB7 promotes tumor proliferation, migration, invasion, and metastasis in several tumor types by interacting with the ERBB protein family. In addition, GRB7 is a potential prognostic marker [25]. The GRB7 protein family includes GRB7, GRB10, and GRB14. These members share conserved domains, such as SH2(C-terminal Src-homology 2), N-terminal proline-rich motif, and central GM (GRB and Mig) region. The conserved regions are involved in protein–protein and protein-lipid interactions. Furthermore, they regulate oncogenic signal transduction [25–27]. According to previous studies, the GRB10 and GRB14 have been shown to play an oncogenic role [12, 15, 28, 29]. And GRB7 has been identified to promote glioma growth and associated angiogenesis in vivo [18]. However, there were no studies that explored the relationship between GRB10 and cell proliferation in gliomas. In the current study, the expression of GRB10 protein was shown to be silenced by shGRB10 RNA. In addition, silencing of GRB10 expression inhibited cell growth, colony formation, invasion, migration, cell cycle, and tumor formation. The results revealed that GRB10 promoted in vivo and in vitro cell growth in glioma.

It is interesting to note that the high expression of GRB14 is shown in breast cancer patients, but the patient with high GRB14 become more sensitively to docetaxel have been reported[30, 31], which may indicate that the patient with high expression of GRB14 isn’t associated with poor prognosis in breast cancer [17]. However, according to data obtained from the TCGA, CGGA, and GEO databases, glioma patients with a low expression of *GRB10* were associated with a longer survival period than patients with a high expression of *GRB10*. Furthermore, RT-qPCR analysis of tumor samples obtained from 68 patients with gliomas revealed that higher histological grades were associated with poorer overall survival (OS). Moreover, the expression of *GRB10* was negatively correlated to the clinical outcomes of glioma patients.

Through GSEA analysis of the high and low expression of *GRB10* groups, we found that the expression of *GRB10* was positively correlated with hypoxia and EMT pathways. Importantly, the hypoxia and EMT pathways played an important role in the development of cancer [32, 33]. In this study, it was confirmed that GRB10 plays an oncogene role in glioma. It is further speculated that GRB10 may promote the progression of glioma by affecting the hypoxia and EMT pathways, but the specific mechanism needs to be further studied.

However, this study had some limitations. First, clinical data were analyzed from samples obtained from 68 patients. leading to biased statistical results. Secondly, the RT-qPCR method was used to detect the expression of *GRB10*, more methods are needed to confirm the protein in glioma tissue. Thirdly, this study did not deeply explore the molecular mechanisms behind glioma cell proliferation, progression, invasion, and migration. Therefore, further studies are required to give insights into the regulatory mechanisms which could potentially be exploited to improve the prognosis of glioma patients.

## Conclusion

This study revealed that high expression of GRB10 could promote tumor formation and progression in gliomas. In addition, higher expression of GRB10 in glioma patients was associated with poorer clinical outcomes.

## Supplementary Information

**Additional file 1: Figure S1.** Unprocessed images of blots. Uncropped images of scanned western blots shown in Figure 3 are provided. **Figure S2.** Unprocessed images of blots. Uncropped images of scanned western blots shown in Figure 5 are provided.

## Data Availability

The datasets used analyzed in the present study are included in the manuscript. These data are available in GEO(https://www.ncbi.nlm.nih.gov/geo/), TCGA (https://portal.gdc.cancer.gov/), and CGGA (http://www.cgga.org. cn) databases. The other data analyzed during this study are available from the corresponding author upon reasonable request.

## Abbreviations

GRB7: Growth factor receptor-bound protein 7
GRB10: Growth factor receptor-bound protein 10
GRB14: Growth factor receptor-bound protein 14
EGFR: Epidermal growth factor receptor
WHO: World Health Organization
OS: Overall survival
RT-qPCR: Quantitative real-time polymerase chain reaction
WB: Western blot
TCGA: The Cancer Genome Atlas
CGGA: Chinese Glioma Genome Atlas
GEO: Gene Expression Omnibus
GSEA: Gene set enrichment analysis
EMT: Epithelial-Mesenchymal transition
