# Supplementary material for: GRB10 is a novel oncogene associated with cell proliferation and prognosis in glioma
Source: Cancer Cell Int. 2022 Jul 5;22:223. doi: 10.1186/s12935-022-02636-5 (PMC9254544; doi:10.1186/s12935-022-02636-5)
Supplement: Supplementary file 1 — Additional file 1: Figure S1. Unprocessed images of blots. Uncropped images of scanned western blots shown in Figure 3 are provided. Figure S2. Unprocessed images of blots. Uncropped images of scanned western blots shown in Figure 5 are provided. [file 12935_2022_2636_MOESM1_ESM.pdf]

Figure S1

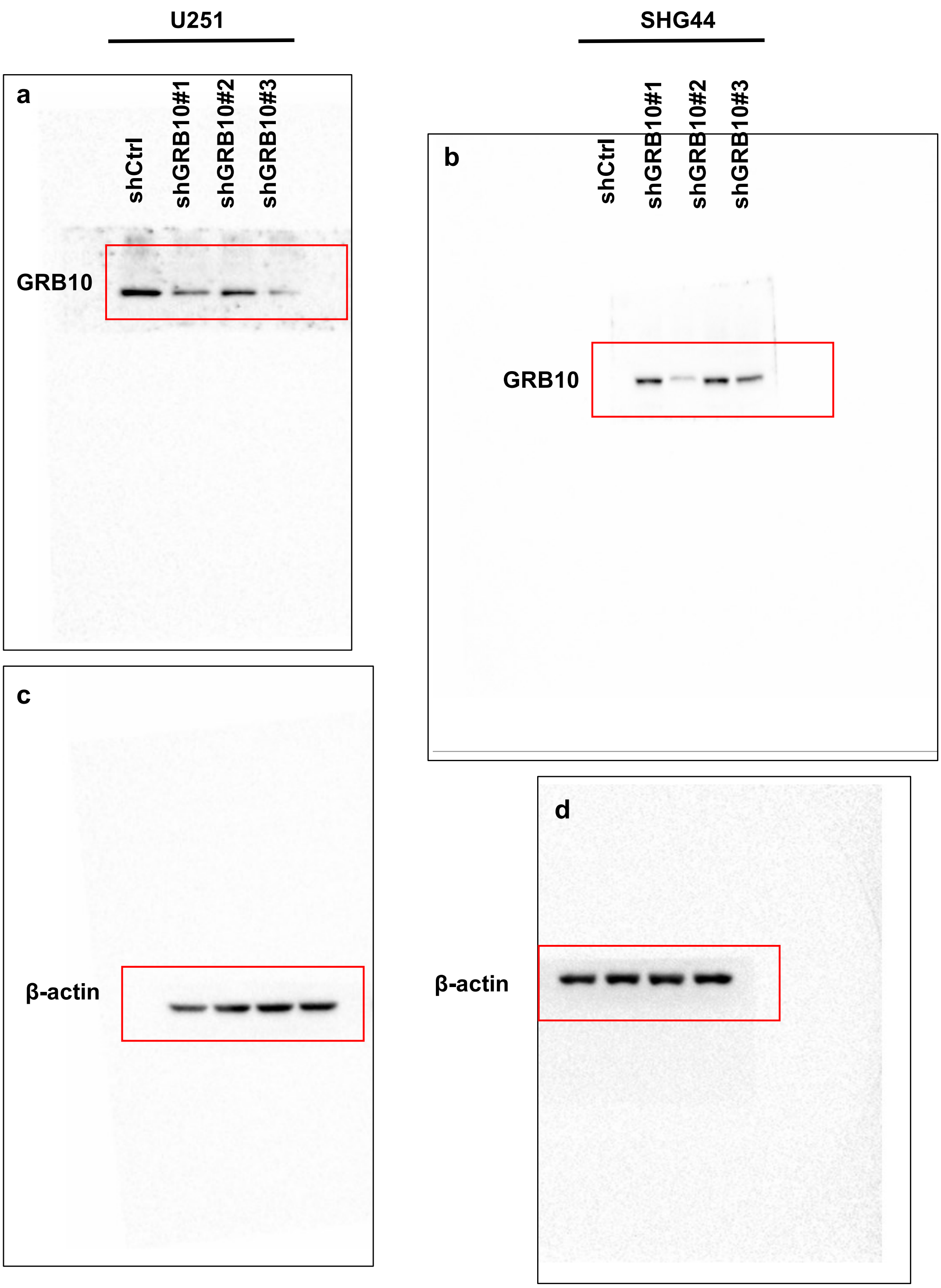

Figure S1 Unprocessed images of blots. Uncropped images of scanned western blots shown in Figure 3 are provided.

Figure S2

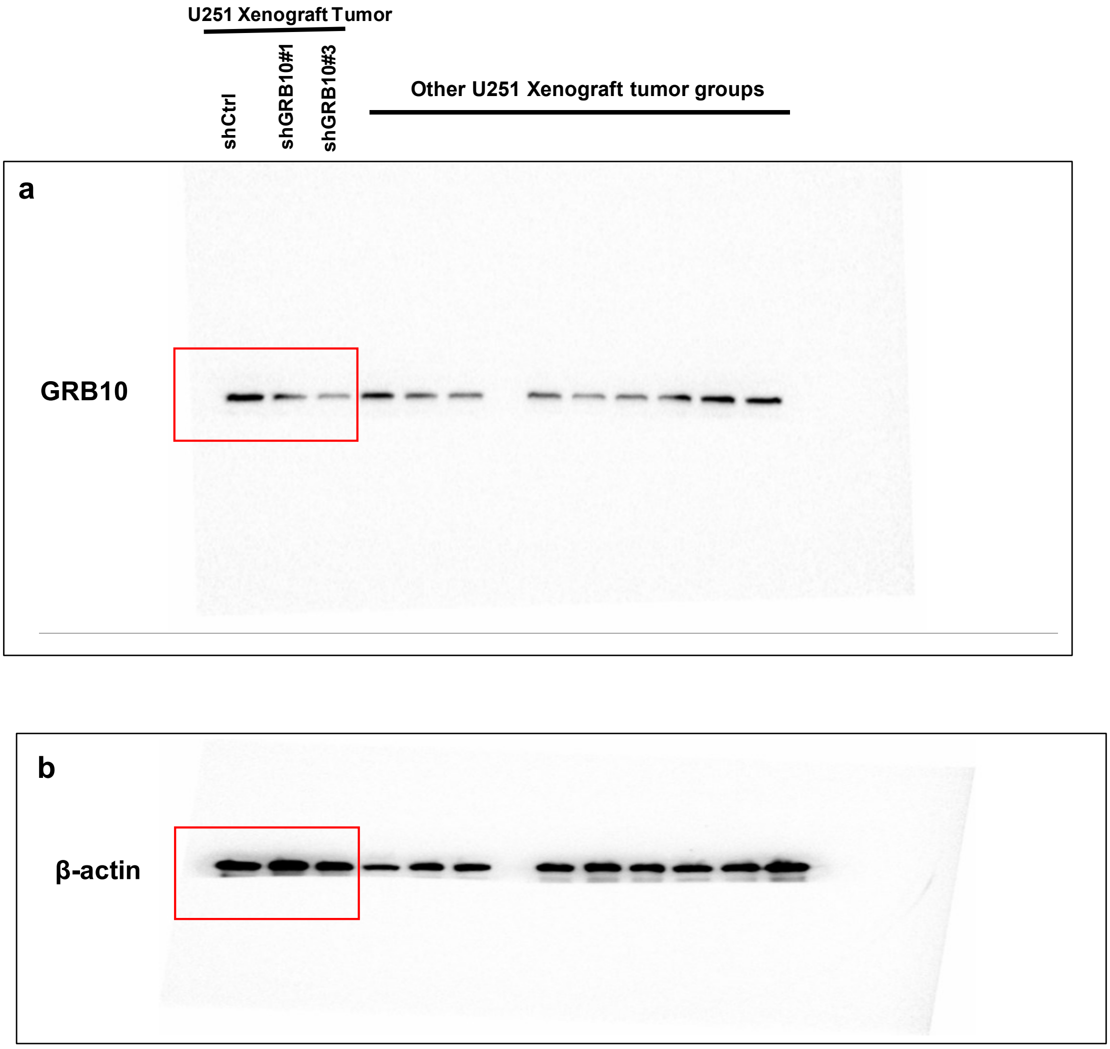

Figure S2 Unprocessed images of blots. Uncropped images of scanned western blots shown in Figure 5 are provided.
